# Recent Advances in Carbon Nanotube-Based Enzymatic Fuel Cells

**DOI:** 10.3389/fbioe.2014.00045

**Published:** 2014-10-24

**Authors:** Serge Cosnier, Michael Holzinger, Alan Le Goff

**Affiliations:** ^1^Département de Chimie Moléculaire (DCM) UMR 5250, Université Grenoble Alpes, Grenoble, France; ^2^Département de Chimie Moléculaire (DCM) UMR 5250, CNRS, Grenoble, France

**Keywords:** biofuel cells, biobatteries, enzymes, nanostructured carbon, modified electrodes, carbon nanotubes, enzyme wiring, laccase

## Abstract

This review summarizes recent trends in the field of enzymatic fuel cells. Thanks to the high specificity of enzymes, biofuel cells can generate electrical energy by oxidation of a targeted fuel (sugars, alcohols, or hydrogen) at the anode and reduction of oxidants (O_2_, H_2_O_2_) at the cathode in complex media. The combination of carbon nanotubes (CNT), enzymes and redox mediators was widely exploited to develop biofuel cells since the electrons involved in the bio-electrocatalytic processes can be efficiently transferred from or to an external circuit. Original approaches to construct electron transfer based CNT-bioelectrodes and impressive biofuel cell performances are reported as well as biomedical applications.

## Introduction

The electrical powering of billions of electronic gadgets like cell phones or computers leads to the production of a countless number of lithium batteries in the environment creating a real problem for human health. Moreover, the ever-increasing depletion of fossil fuels and the need for clean methods of producing electricity have stimulated the emergence of new sources of sustainable and renewable energy without greenhouse gas emissions or environmental pollution. Among these clean alternative sources, the production of electrical energy thanks to biofuel cells, a subcategory of fuel cells, is a rapidly growing field. In particular, enzymatic fuel cells that convert chemical energy into electrical energy by catalytic reaction of the enzymes, is one of the most common and studied configuration (Barton et al., 2004; Cracknell et al., 2008; Zayats et al., 2008; Willner et al., 2009; Ivanov et al., 2010; Leech et al., 2012; Katz and MacVittie, 2013; Cosnier et al., 2014). These biofuel cells use redox enzymes for the specific oxidation of fuels (alcohols, hydrogen, lactate, sugars such as glucose, fructose, lactose, or cellobiose) at the anode and the reduction of oxidizers (O_2_, H_2_O_2_) at the cathode in order to generate electric power (Meredith and Minteer, 2012). A vast majority of these biofuel cells produce electric power from the electro-enzymatic degradation of glucose and oxygen. Compared to hydrogen or methanol fuel cells, sugars like glucose present the unique advantage of being a perfect energy storage compound in many living organisms and have absolutely no toxicological, explosive or flammable risks. Taking into account that catalysts, fuels, and products are biodegradable, the inherent ecological aspect of the biofuel cells compared to fuel cells should be noted. Fuel cells require catalysts based on precious metals or transition metals such as nickel, gold, silver, rhodium, ruthenium, palladium or chromium, or alloys such as Raney nickel (Zhang, 2008).

The main application of biofuel cells is to design devices whose power and size will be compatible with a use as portable source of energy (miniature generators of low power for mobile phone or GPS). Combined with conventional batteries, these biosystems will also be able to ensure a recharging of the batteries and a standby mode for electronic equipment. Owing to the presence of some fuels such as glucose and lactate in physiological fluids, another major motivation for the development of biofuel cells concerns the production of electricity from human body. Two approaches are in constant development: enzymatic fuel cells implanted in the body and using glucose present in the blood or interstitial fluids and non-invasive fuel cells using lactate present in human perspiration. The objectives are for the former to power implanted medical devices like cardiac pacemakers, muscle stimulators, neurological stimulators, cochlear implants, drug pumps, sensors, while those for the latter are to harvest energy from human for powering wearable electronics (Jia et al., 2013; Katz and MacVittie, 2013; Cosnier et al., 2014).

Although the first example of a biofuel cell has been reported in the 60s (Yahiro et al., 1964), the development of such promising devices has remained scarcely investigated until the late 90s. Since the early 2000s, tremendous advances have been achieved in the field of biofuel cells as evidenced by the exponential increase of scientific publications devoted to this topic. This results from the removal of technological barriers directly related to profound progress made in the field of electrochemical biosensors like the design of new materials and procedures for immobilization and electrical connection of enzymes (Polsky et al., 2001; Ronkainen et al., 2010; Samanta and Sarkar, 2011).

In particular, the development of biointerfaces has triggered enormous attention in the field of energy conversion. Taking into account that the bioelectrode activity was related to the activity of the wired enzymes, three-dimensional structures were designed to enhance the specific surface of the conductive substrate and the immobilized amount of enzymes and redox mediators serving as electron shuttle between enzymes and the electrode surface. In this context, numerous efforts have been focused in the production of novel biomaterials based on aerogels (Wen et al., 2014), osmium-derivatized polymers (Shaoa et al., 2014), redox hydrogels (Flexer and Mano, 2014; Plumeré et al., 2014), inorganic clays (Zebda et al., 2011a), and conductive nanomaterials (Willner et al., 2006; Holzinger et al., 2012). For instance, deposited hydrogel films based on osmium-containing metallopolymer that are permeable to water-soluble chemicals, were widely employed by Mano’s and Heller’s group for designing enzymatic fuel cells. These conducting polymer hydrogels were thus applied to the entrapment of enzymes (glucose oxidase, glucose dehydrogenase, bilirubin oxidase, laccase) onto platinum microelectrodes and nanostructured scaffolds of carbon fibers grown by chemical vapor deposition (Soukharev et al., 2004; Little et al., 2011; Flexer et al., 2013). In addition, the modulation of the tethering of redox centers to the polymer backbone was investigated for optimizing the enzyme wiring (Forster et al., 2003). Moreover, the stabilization of the bioarchitectures was envisioned by photoinitiated polymerization of poly(ethylene-glycol) diacrylate as outer layer (Suraniti et al., 2011).

Owing to the intense research activity in this field, we aim not to give a complete coverage of biofuel cells literature involving 3D constructs, but rather to review briefly the recent strategies employed with carbon nanotubes (CNT) for enzyme immobilization and their wiring.

## Enzymatic Fuel Cells Based on Carbon Nanotube Deposits

Within the vast number of available nanostructured materials and nano-objects, CNTs exhibit, between others, nanowire morphology, biocompatibility, and excellent conductivity (Dai, 2002; Smart et al., 2006). Due to their geometry, CNT presents an impressive high specific surface of more than 1000 m^2^/g constituting thus an attractive building block for the construction of highly porous three-dimensional nanostructured CNT electrodes (Peigney et al., 2001). These particularities confer to nanotubes a pivotal role for designing electrochemical biosensors and biofuel cells. Furthermore, the possibility to add appropriate functionalities via organic functionalization enabled optimal tuning of such nanostructured electrodes by attaching specific (redox) sites for fixing proteins or catalyzing electrochemical reactions with enzymes or coenzymes.

**Figure 1:**
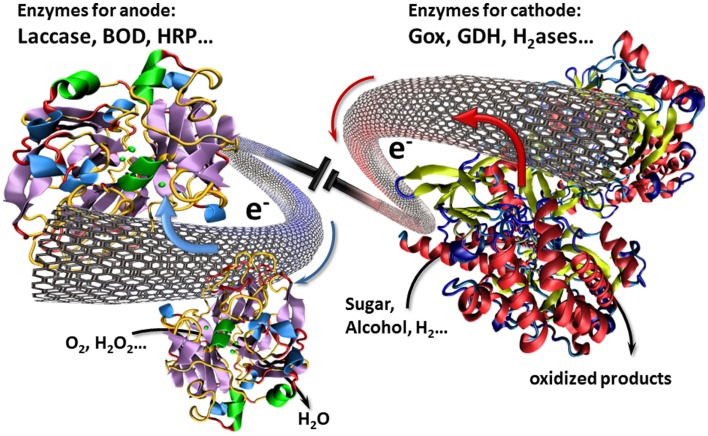
**Illustration of a biofuel cell setup using CNTs as nanowires for the transfer of electrons involved in the electrocatalytic redox reactions**.

In this context, CNTs have played an important role for interfacing enzymes with electronic circuitry. In particular, these CNT can establish an electrical communication with enzymes via their intrinsic conductivity or via an electron transport to enzymes ensured by electron hopping between immobilized redox centers. With regard to their nanoscale size, CNTs can approach in close proximity the prosthetic site of enzymes and hence achieve a direct electrical wiring between enzymes and the bulk electrode (Figure 1). As a consequence, electrodes modified by CNTs have aroused widespread attention in the design of biofuel cells (Holzinger et al., 2012).

A significant step in the development of bioelectrodes was thus described in 2010 by Cosnier and coworkers who have patented a new approach to create “ex nihilo” bioelectrodes by compressing a powder mixture of CNT and enzyme (Cosnier et al., 2010). This method of bioelectrode fabrication that leads to mechanically stable disks was applied to the development of glucose biofuel cells. The compression of CNTs with glucose oxidase and laccase enables direct electron transfer (DET) between the enzymes and the CNT matrix thus shuttling the involved electrons of the oxidation of glucose and the reduction of oxygen to an external circuit. The resulting mediatorless enzymatic fuel cell delivered a remarkable high open-circuit voltage (0.95 V) and an impressive maximum power density of 1.25 mW cm^−2^ (1.66 mW mL^−1^, 1.85 mW g^−1^) and 0.14 mWh cm^−2^ under continuous discharge in a 50 mmol L^−1^ glucose solution (Zebda et al., 2011b). Surprisingly, it appears that the glucose biofuel cell performance is limited by the bioanode although the glucose concentration was markedly higher than that of O_2_. This phenomenon may be ascribed to a more efficient electrical wiring of laccase than glucose oxidase, which has its redox active prosthetic groups deeply located inside the protein shell. The occurrence of a DET process at the bioanode was investigated by cyclic voltammetry experiments. In the absence of glucose, a reversible system was observed at E1/2 = −0.46 V vs. SCE (Reuillard et al., 2013). The latter was attributed to the electroactivity of the FAD, the prosthetic group of glucose oxidase. In addition, a weak electrocatalytic anodic current appeared in presence of glucose (150 mM). However, the origin of this system remains questionable and could be due to a partial opening of the protein, the stability of this redox signal eliminating the assumption of a total release of the FAD from the protein. The weak intensity of the glucose oxidation at this potential (−0.4 V) reflects that only few CNTs could get in sufficient close contact to glucose oxidase by this compression method, to regenerate the enzyme and hence, a low percentage of the immobilized glucose oxidase was efficiently connected. As a consequence, a new configuration of compressed bioanode based on mixed DET and mediated electron transfer (MET) was reported. The concept is based on simple addition of naphthoquinone as redox mediator to the glucose oxidase/CNT mixture before compression, leading to its immobilization within the resulting disk. Thanks to its small size, this redox mediator serves as electron shuttle capable to collect the electrons from the glucose oxidase and to transfer them to the CNT matrix. By using the same biocathode, the power density of the completed biofuel cell increased to 1.54 mWcm^−2^ (1.92 mW mL^−1^, 2.67 mW g^−1^) reflecting thus an improved wiring of the entrapped enzymes (Reuillard et al., 2013). This biofuel cell setup with the modified bioanode is also able to constantly deliver 0.56 mWh cm^−2^ under long-term discharge. Owing to the high amount of immobilized glucose oxidase, an attractive storage stability of the enzymatic fuel cell stored in phosphate buffer at room temperature was observed, the open-circuit voltage decreasing from 0.76 to 0.5 V after 6 months.

For the electrochemical storage of energy, CNTs are often employed as basis material for the construction of high performance supercapacitors with extremely short charging time and high capacitance. The latter can act as the bridge between batteries and conventional capacitors due to their properties to store high energy densities combined with rapid charge/discharge cycles. As a consequence, compressed disks of enzymes-CNTs were used as supercapacitors and electrodes for a biofuel cell setup. It was expected that the possibility to recharge supercapacitors with an internal energy source could thus represent a significant improvement for the performance of biofuel cells. This hybrid supercapacitor/biofuel cell enables high-power discharge cycles, the CNT disks being continuously recharged through the biocatalytic energy conversion in neutral buffered glucose solutions (Agnès et al., 2014). In addition, this hybrid device presents an attractive operational stability delivering 40,000 constant pulses of 2 mW for 10 ms every 10 s at least for 5 days.

On the other hand, CNTs can form flexible and high conductive sheets called buckypapers. Among different ways to form buckypapers, the most common approach is vacuum filtration of CNT dispersions where important factors are the purity and the graphitization of CNT and their homogeneous dispersion. For instance, efficient biocathodes for biofuel cells were prepared by adsorbing laccase on multiwalled CNTs buckypaper fabricated by dispersion in water and filtration on nylon membrane filter. These bioelectrodes catalyze the reduction of oxygen with an open-circuit potential of 0.64 V vs. SCE providing a current density of about 0.43 mA cm^−2^ at 0.5 V (Hussein et al., 2011). However, commercial mass produced multiwalled CNTs generally have highly defective outer walls, which prevent the formation of buckypapers without additives (Zhang et al., 2013). To overcome these issues, an innovative approach lies in the crosslinking of CNTs by organic spacers bearing several specific groups capable of generating covalent or non-covalent bonds with CNT walls. In particular, the non-covalent modification of CNT coatings was attempted with pyrene derivatives enabling π–stacking interactions with CNT wall. Buckypaper electrodes with enhanced mechanic stability were formed using a classical filtration technique of a CNT suspension in presence of a bis-pyrene crosslinker containing the redox mediator 2,2′-Azino-bis(3-ethylbenzothiazoline-6-sulfonic acid (ABTS) (Bourourou et al., 2014). Beside the formation of reinforced buckypaper, this bis-pyrene-ABTS assures the MET to laccase. The resulting buckypaper electrodes were thus applied to oxygen reduction using laccase in solution. For this setup, the redox buckypaper electrodes demonstrate high performances with maximum currents up to 1 mA cm^−2^ ± 40 μA cm^−2^. Buckypaper-based biocathodes were designed for air-breathing conditions using laccase or bilirubin oxidase as catalysts reaching up to 755 ± 39 mA cm^−2^ at 0.3 V vs. Ag/AgCl (Babanova et al., 2014) and 0.5 mA cm^−2^ at zero potential vs. Ag/AgCl (Lau et al., 2012), respectively.

## New Advances in Biocathodes Based on CNT Constructs for Oxygen Reduction

While sugars and alcohols can reach sufficient molar concentrations in water, the low concentration of oxygen may be a limiting factor for the performances of the enzymatic fuel cell. Over the past several years, we have witnessed an exponential growth in publications on optimizing biocathodes mainly based on laccase for oxygen reduction. For instance, among various techniques employed to entrap laccases in CNT matrices, an original water-induced shrinkage of a free-standing MWCNT-forest film has shown remarkable performances for the enzyme entrapment and the direct wiring of laccases (Miyake et al., 2011a). Taking into account that laccase exhibits a hydrophobic cavity near its peripheral T1 copper center, the possibility to orientate and to wire this enzyme during its immobilization may increase the electron transfer rate, and therefore, the catalytic current. This hydrophobic domain even leads to spontaneous oriented adsorption on carbon electrodes. Rubenwolf et al. took advantage of this effect and designed a buckypaper-based cathode where the catalyst could be renewed by changing the enzyme containing electrolyte where clearly enhanced biocathode lifetimes could be reached (Rubenwolf et al., 2012). The efficiency of this principle for lifetime elongation was confirmed with multicopper enzyme containing crude fungal culture supernatants (Sané et al., 2013). Similarly, multicopper oxidases from enzyme-secreting recombinant planktonic microorganisms have been used, though with these a lifetime elongation has not yet been (Sané et al., 2014).

**Figure 2:**
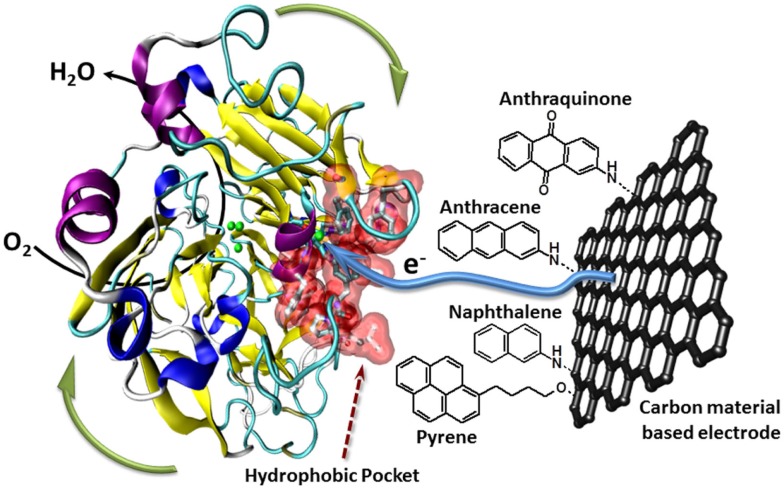
**Schematic presentation of the oriented immobilization of laccase via supramolecular interactions between the laccase’s hydrophobic pocket and polyaromatic hydrocarbons like anthraquinone, anthracene, nathpthalene, or pyrene attached to carbon material based electrodes**.

Furthermore, the Armstrong’s group has modified electrodes with aryldiazonium derivatives having π-extended hydrophobic groups such as anthraquinone, anthracene, naphthalene, or chrysene to graft and connect laccase (Blanford et al., 2009). Several examples report the efficient immobilization, orientation, and wiring of laccase using polyaromatic hydrocarbons such as anthracene or naphthalene (Meredith et al., 2011). More recently, the ability of anthraquinone to immobilize and orientate laccase was optimized using a bis-anthraquinone-pyrene derivative whose geometry only authorizes a partial π-stacking on CNTs facilitating thus the laccase binding (Bourourou et al., 2013). The use of CNT-based electrodes allows the achievement of high catalytic currents of more than 1 mA cm^−2^. Another strategy consists in functionalizing CNTs by electrochemical polymerization of organic films. An electrogenerated poly(pyrrole–pyrene) allows thus the covalent immobilization of laccase via its interaction with polymerized pyrene, leading to a high performance biocathode with a catalytic current density of 1.85 mA cm^−2^ at 0.3 V (vs. SCE) in oxygen-saturated solution. In addition, these electrodes showed also excellent stabilities under continuous discharge (Lalaoui et al., 2013) (Figure 2).

## Enzymatic Fuel Cells Harvesting Energy from Living Organisms

Taking into account that glucose and lactate are present in many living organisms, some biofuel cells were designed for harvesting energy from fruits, small insects, and animals. The main configurations of enzymatic fuel cells involved bioanodes based on glucose oxidase, glucose dehydrogenase, or lactate oxidase and biocathodes based on copper oxidases such as laccase, tyrosinase, or bilirubin oxidase. This concept was initiated by Mano et al. who implanted microbioelectrodes based on osmium redox hydrogels, in a grape obtaining thus 2.4 μW at 0.54 V (Mano et al., 2003). In 2010, the first example of an enzymatic fuel cell totally implanted in a mammal (inside the retroperitoneal space of a rat) was described (Cinquin et al., 2010). This biofuel cell was based on compressed graphite-based disks entrapping redox mediators and tyrosinase and glucose oxidase at the cathode and anode, respectively. Although this work demonstrated the possibility of harvesting energy by a biofuel cell implanted inside a mammal, the open-circuit voltage and power density were far below the levels required to supply implanted biomedical devices. Different examples of biofuel cells partially implanted in the abdomen of an insect (a cockroach species) (Rasmussen et al., 2012) or directly in blood in a vein of a rabbit (Miyake et al., 2011b) or in the jugular vein of a rat (Sales et al., 2013) were more recently reported. However, these biofuel cells did not show sufficient performance to power electronic systems. Nevertheless, Mao and coworkers presented an original concept where a glucose biofuel cell itself acts as sensor to monitor the glucose concentration *in vivo*. Here, the output cell voltage is related to the glucose concentration of the rat’s brain fluid, conducted through an external microfluidic circuit (Cheng et al., 2013).

With the aim to improve the performance of the implanted enzymatic fuel cells and, contrarily to the initial concepts, researchers conceived biofuel cells based on CNT. Thus buckypaper electrodes composed of compressed multiwalled CNT were modified by pyrene-butanoic acid via π-stacking interactions. These functionalized buckypapers were subsequently functionalized by chemical grafting of PQQ glucose dehydrogenase at the bioanode and laccase at the biocathode enabling a DET between enzyme and CNT electrodes. The validity of this approach was initially demonstrated through the implantation of biofuel cell in snail and more recently on the surgically exposed cremaster tissue of a rat (Halámková et al., 2012; Andoralov et al., 2013). Similar enzyme-buckypaper electrodes were employed for biofuel cells implanted in clams and lobsters connected in series (Szczupak et al., 2012; Southcott et al., 2013). Although lobsters are crustaceans, which are arthropods and are therefore distant from mammals, this work represents a breakthrough in the field of biofuel cells by demonstrating that an implanted biofuel cell can harvest enough energy from the compounds present in physiological fluids to power a pacemaker, an electrical motor, or a watch. As demonstrated by the Katz’s group, the biofuel cell voltage is limited but its interfacing with microelectronic circuits can lead to devices that can deliver a voltage of several volts. Beside this, a biofuel cell based on compressed CNT-enzymes disks and wrapped in dialysis bag and Dacron bag, was successfully implanted by surgical insertion into the retroperitoneal space of rats. This biofuel cell delivered *in vivo* an open-circuit voltage of 0.85 V. These measurements show that using a step-up converter circuit, the biofuel cell was able to power in physiological conditions, a Light Emitting Diode and a thermometer (Zebda et al., 2013). However, the development of implanted biofuel cells must meet the criteria of biocompatibility, sterilization, and long operational stability. The insertion of a biofuel cell in animals and in particular human bodies requires invasive and strict procedures. In this context, another strategy exemplified by Wang’s group, lies in the development of non-invasive biofuel cells that can harvest energy from metabolites present on the epidermis or even in subcutaneous level (Jia et al., 2013; Valdés-Ramírez et al., 2014).

## Conclusion

Despite impressive results in energy harvesting using sugars and oxygen as fuels, further progress is needed as this technology becomes competitive with lithium batteries, which are currently used for electronic devices. For example, the state of the art fuel cell glucose normally offers several hundred microwatts up to few milliwatt at about 0.4–0.5 V with stabilities up to several weeks. This is not sufficient to power electronic devices without step-up converters. Nevertheless, owing to the wide range of available additives, all with their own specific properties, it clearly appears that the combination of various compounds (polymers, nanoparticles, redox mediators, crosslinking agents) with CNTs in various forms (buckypaper, pellets, forest, etc.) is a promising way to increase the performances of enzymatic fuel cells.

## Conflict of Interest Statement

The authors declare that the research was conducted in the absence of any commercial or financial relationships that could be construed as a potential conflict of interest.
